# Segmental folding of chromosomes: A basis for structural and regulatory chromosomal neighborhoods?

**DOI:** 10.1002/bies.201300040

**Published:** 2013-07-05

**Authors:** Elphège P Nora, Job Dekker, Edith Heard

**Affiliations:** 1 Institut CurieParis, France; 2 CNRS UMR3215Paris, France; 3 INSERM U934Paris, France; 4 Program in Systems Biology, Department of Biochemistry and Molecular Pharmacology, University of Massachusetts Medical SchoolWorcester, MA, USA

**Keywords:** chromatin domains, chromatin folding, chromosome conformation capture (3C), long-range transcriptional regulation, regulatory landscapes, topologically associating chromosome domains

## Abstract

We discuss here a series of testable hypotheses concerning the role of chromosome folding into topologically associating domains (TADs). Several lines of evidence suggest that segmental packaging of chromosomal neighborhoods may underlie features of chromatin that span large domains, such as heterochromatin blocks, association with the nuclear lamina and replication timing. By defining which DNA elements preferentially contact each other, the segmentation of chromosomes into TADs may also underlie many properties of long-range transcriptional regulation. Several observations suggest that TADs can indeed provide a structural basis to regulatory landscapes, by controlling enhancer sharing and allocation. We also discuss how TADs may shape the evolution of chromosomes, by causing maintenance of synteny over large chromosomal segments. Finally we suggest a series of experiments to challenge these ideas and provide concrete examples illustrating how they could be practically applied.

## Introduction

**Figure 1:**
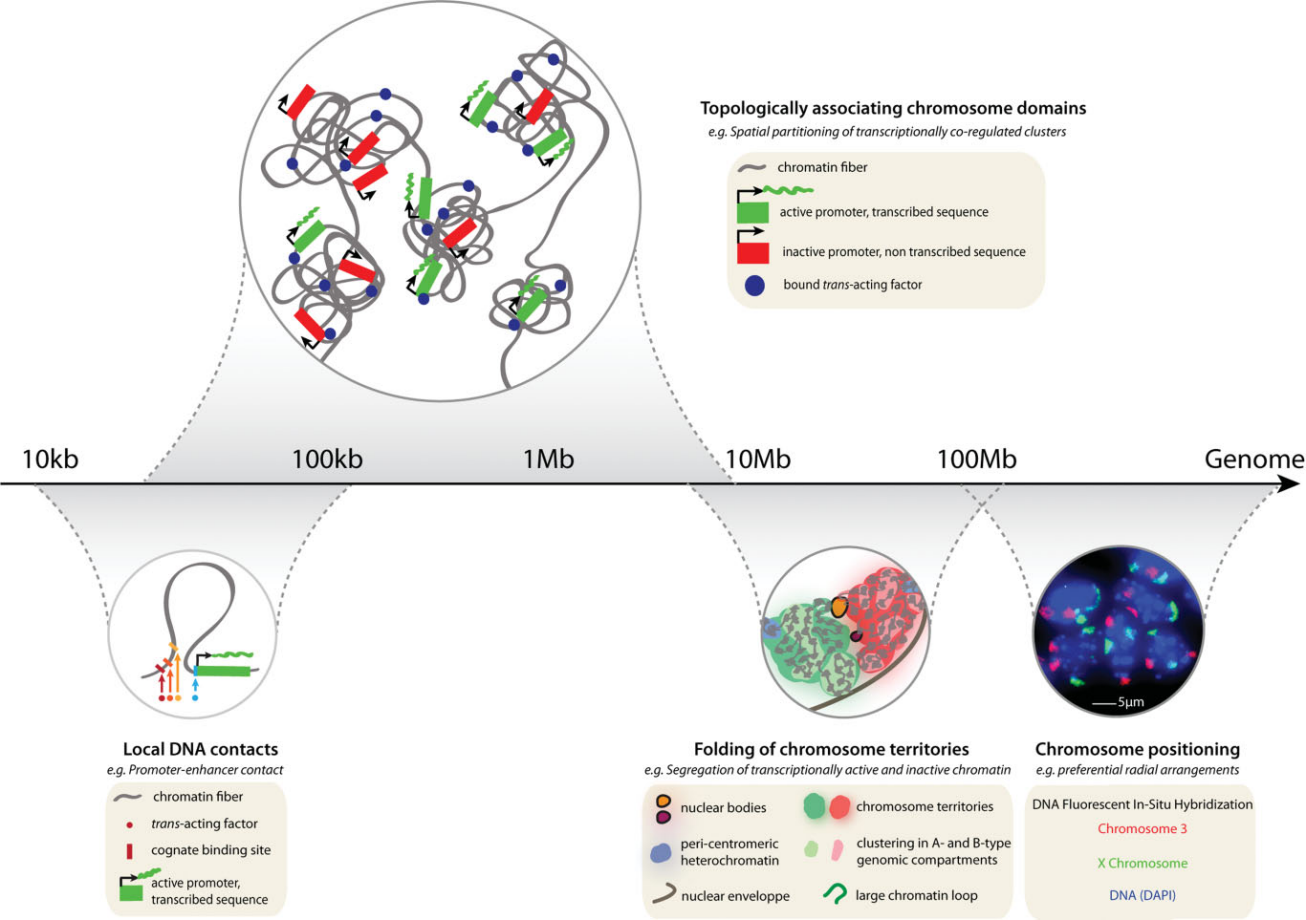
Scales of genome architecture. The folding rules of the genome are hierarchical, with different principles applying at each scale. Local DNA contacts, by means of looping or other configurations, play a central role in controlling the communication between enhancers and promoters. These contacts most often take place within TADs, which define what groups of sequences cluster together. Relative arrangement of TADs then shapes the chromosome territory, within which transcriptionally competent regions are typically segregated away from the transcriptionally inert ones. Within the nucleus each chromosome tends to occupy a preferential radial position, sometimes depending on the cell type.

In recent years considerable progress has been made to further our understanding of chromosome organization. Blossoming applications of Chromosome Conformation Capture (3C) have played a central role in fostering these advances 1, enabling the exquisite dissection of local chromatin folding at scales ranging from tens to hundreds of kilobases (kb), beyond the resolution of optical microscopy (see 2 for review). Seminal studies employing 3C have for example formally demonstrated the existence of physical contact between regulatory elements and their remote target promoters 3 (Fig. 1).

High-throughput 3C approaches 2 have allowed researchers to study higher-order chromosome architecture, at scales ranging from tens to hundreds of megabases (Mb). At these scales microscopy can readily be used and has supported 3C results, revealing the tendency of transcriptionally active fractions of the genome to spatially segregate from inactive ones 4–5 – see Box 1. These experiments have also provided a way of assessing the relative positioning of chromosomes and revealed the ability of some chromosomal regions to contact each other in *trans* 5–10 (Fig. 1).

Box 1

**Hierarchical folding of the chromosome territory**

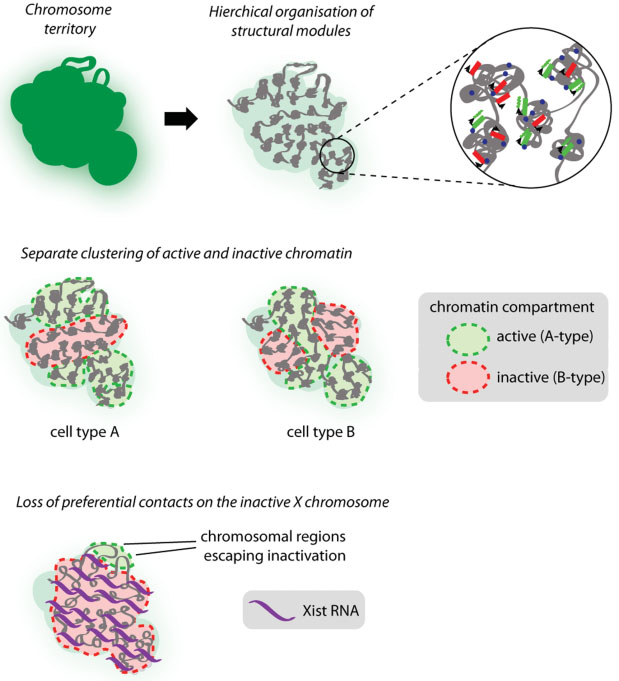

The chromosome territory represents the relative positioning of topologically associating domains (TADs) in the three-dimensional space of the nucleus. The relative arrangement of TADs may differ between cell-types, but is generally related to their transcriptional activity, so that groups of active TAD coalesce to form the A-Type genomic compartment while the inactive TADs are segregated away in the B-Type compartment 4–5 – see 72 for review. The only reported situation where folding into TADs is impaired is the case of the inactive X chromosome in female cells, where long-range chromosomal contacts appear to be more pervasive and less specific both within and between TADs 13–58. Such loss of preferential contacts is due to the action of the Xist long non-coding RNA, independently of its ability to repress transcription 58.

In spite of these achievements, chromosomal organization at the sub-Mb scale has remained elusive – partly because of the technical compromise between genomic resolution and coverage imposed by 3C technologies. These limitations are starting to be overcome thanks to the increase in sequencing depth of Hi-C data 5 and the up-scaling of Chromosome Conformation Capture Carbon-copy (5C) to span large regions 11. Indeed, important recent progress has been made in our understanding of chromosome architecture around the Mb-scale, both in mammals 12–13 and *Drosophila* 14–15.

These studies have revealed that chromosomes are segmented into large domains, encompassing tens of kb in *Drosophila* and up to hundreds of kb in mammals, within which physical contacts, as measured by 3C, occur much more frequently than between domains (for review see 16–17). In a study focused on part of the mouse X-chromosome, direct imaging by DNA fluorescence in situ hybridization (FISH) confirmed that sequences belonging to the same domain preferentially fold together, but also highlighted that the shape, compaction, and extent of spatial separation of these domains can be highly variable from cell to cell 13. To what extent these domains correspond to the Mb-wide structures previously observed by microscopy 18 remains to be investigated. Inter-domain chromosomal contacts are observed by 3C and FISH, but at lower frequency than intra-domain interactions. These observations have been proposed to reflect the physical packaging of DNA into discrete chromosomal modules (Fig. 1), which have been termed “topologically associating domains” (TADs) 13, “topological domains” 12, or “physical domains” 15.

Based on these recent findings, we present here a series of testable hypotheses concerning how chromosome folding into TADs may relate to, and possibly influence, several aspects of chromosome structure and function, as well as evolution. We also discuss how such knowledge can be practically extended to fields beyond basic chromosome biology, such as transgenesis approaches as well as human genetics. Our discussion mainly concerns mammalian genomes, but also feeds on supporting observations made in *Drosophila*, which have been recently reviewed elsewhere 19.

## Chromosome structure

### TADs and other chromatin domains

TADs partition chromosomes into neighborhoods of approximately 1 Mb in mammals 12–13 and 100 kb in *Drosophila* 14–15. This level of chromosomal segmentation appears to be lacking from the much smaller chromosomes of *S. cerevisiae* and *S. pombe* yeasts 20–21. The scale at which TADs occur raises the question of their relationship with other domain-wide chromosomal features that have previously been described 22. Indeed, it is well established that chromosome structure is not homogeneous 23–24 (see 25 for review). Genome-wide mapping of DNA-binding protein occupancy has shown that some factors tend to associate with chromatin over Mb-sized blocks, rather than as focal peaks (see 26 and 16 for review). The post-translationally modified histones H3K9me2, H3K9me3, and H3K27me3 are among such factors as they can each mark distinct chromosomal domains 27,28. Enzymes responsible for depositing these marks do not recognize DNA in a sequence-specific manner and the mechanisms that control the localization of these chromatin domains along chromosomes is not fully understood. The fact that many boundaries of such chromatin domains align remarkably well with TAD boundaries, suggests a mechanistic link between these two types of chromosome domains.

**Figure 2:**
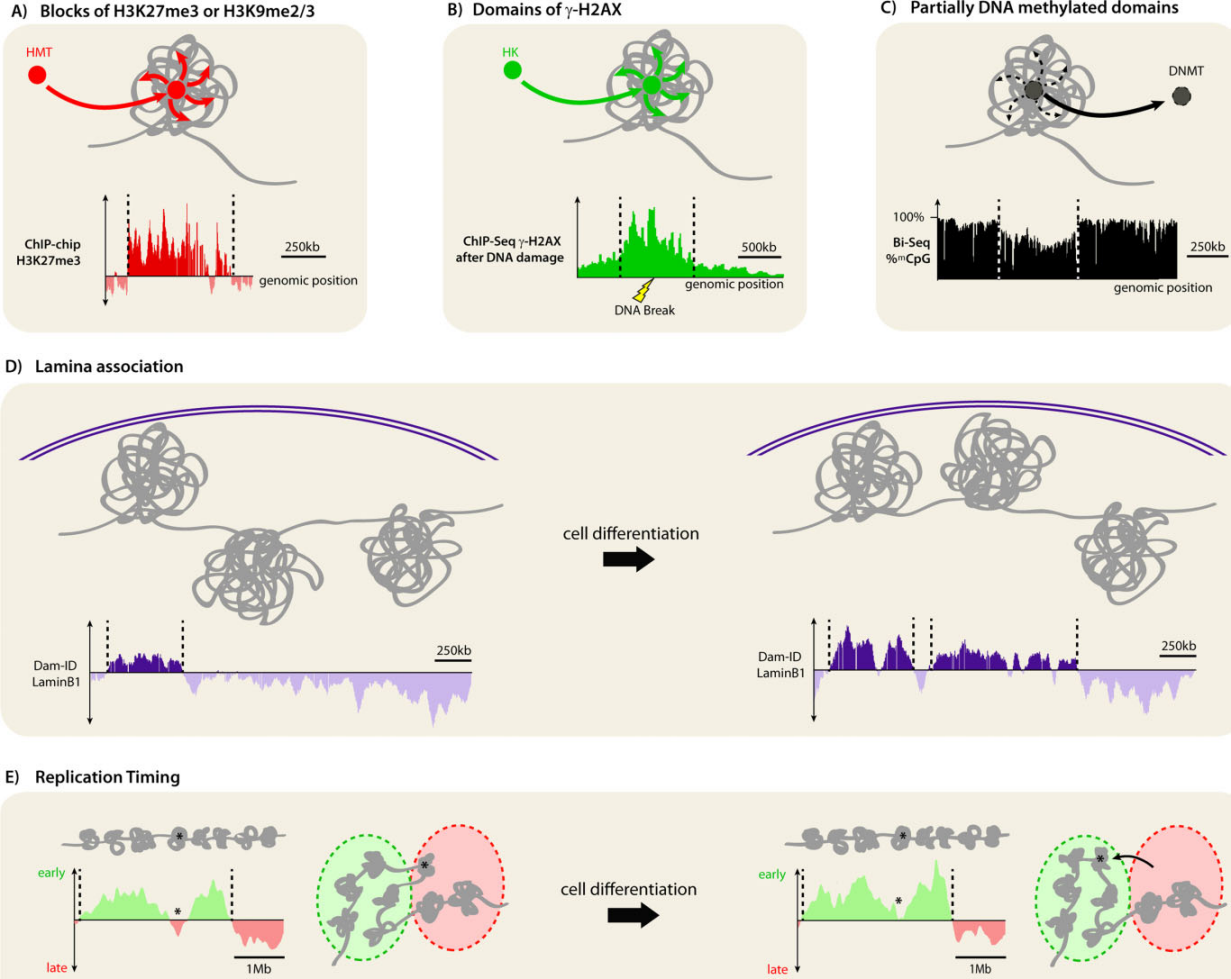
The Link between TADs and domain-wide chromatin features. The positions of TADs along chromosomes align with several types of domain structures that suggest a possible mechanistic link between the two. We hypothesize that these mechanisms can rely on local three-dimensional diffusion sites A: histone methyl-transferases (HMT) or B: histone kinases (HK) from primary recruitment sites. C: Mechanistic cross-talk, such as for example between the polycomb and DNA methyl-transferase machineries (DNMTs), could explain the indirect correspondence with other types of domains such as partially DNA methylated domains. TADs may therefore represent modular units of chromosomes that can assume different structural fates. For example D: LADs are found to correspond to TADs, and their developmental dynamics could be explained by the repositioning of TADs to or away from the nuclear envelope. E: Similarly, even though most replication domains overlap multiple TADs, changes in timing during cell differentiation typically involve TAD-sized regions. Data are from published sources for H3K27me3 70, γH2AX 32, Bisulfite (Bi)-seq 71, LaminB1 36 and replication timing 39, and TADs 12–13.

How could such a connection arise? Some studies have suggested that chromatin composition, and especially the presence of H3K27me3, can influence its local conformation 30. Chromatin composition does not appear to be what drives chromosomes folding into TADs however, as only a few TADs harbor distinctive domain-wide chromatin signatures. Furthermore, the positioning of TADs along the chromosome is not affected when the chromatin composition of these blocks changes, either naturally during cell differentiation, or artificially by disruption of histone modifying enzymes 13. The question is therefore how such a coincidence in the positioning of chromatin blocks and TADs along chromosomes occurs. It has been suggested that limiting three-dimensional diffusion of chromatin-modifying enzymes plays an important role in assembling specific chromatin domains, in particular at peri-centromeric heterochromatin 31. Based on these ideas we propose that the spatial clustering in TADs may actually determine the genomic span of chromatin domains throughout the genome (see Fig. 2 and corresponding legend). In this context we discuss observations that argue that spatial folding of chromosome domains into TADs may underlie the domain-wide nature of several chromatin features.

### A role in the spreading of chromatin modification?

Upon DNA double-strand break induction H2AX becomes rapidly phosphorylated around the damage site, forming nuclear foci that typically contain thousands of histone molecules. It appears that at least some of these γ-H2AX domains align with TADs 12–32, suggesting that histone phosphorylation spreads across a genomic distance that corresponds to the TAD inside which the break has occurred. We hypothesize that such spreading effects actually reflect the local diffusion of chromatin modifying enzymes, in three-dimensions, from their primary recruitment sites into adjacent chromosomal regions that are physically packaged in the same TAD (Fig. 2A and B). Importantly, this would provide a complementary mechanism to the generally envisioned linear nucleosome-to-nucleosome propagation of histone-modifying enzymes. Indeed, models so far have fallen short of explaining how spreading can take place over Mbs, or the existence of non-modified nucleosomes within such chromatin domains 32–33. The link between folding into TADs and chromatin composition may be reflected directly or indirectly on other domain-wide features of the epigenome. For example, the presence of H3K27me3 appears to be molecularly linked to the absence of DNA methylation and thus a gain of domain-wide H3K27me3 across a TAD, for example during tumorigenesis 34, may lead to the TAD becoming globally DNA hypomethylated (Fig. 2C).

### Stable TADs of changing chromatin states

Positioning of TADs along chromosomes appears to be largely invariant between cell types 12–13. Although a subset of TAD boundaries clearly seem cell-type specific, the current resolution of Hi-C hampers the precise determination of the number of tissue-specific TADs 12. Although the global positions of TADs across chromosomes during development are preserved, in contrast chromatin blocks – for example those harboring similarly post-translationally modified histones – are much more dynamic. This raises the possibility that chromatin folding into TADs can represent a structural basis onto which various epigenomic features are then overlaid in a domain-wide fashion. Consistent with this is the genomic distribution of the large genomic neighborhoods that associate with the nuclear lamina 35. These lamina-associated domains (LADs) are largely constant between cell types 36 and typically align with TADs 12–13. The few exceptions that are tissue-specific apparently correspond to the relocation of pre-existing TADs to the nuclear lamina (Fig. 2D). Altogether this raises the possibility that chromosome segmentation into TADs allows a certain degree of modularity in the organization of the epigenome at the Mb-scale. Some TAD boundaries are however clearly tissue-specific and analysis of more cell types will help in understanding the origin and the consequences of such variations. Given these considerations the segmental organization of chromosome folding into TADs may explain the regional nature of the changes in chromatin states observed during both normal 29–36 and pathological 37–38 cell differentiation.

### TADs: Units of replication timing shifts?

The time-point at which DNA replication initiates during S-phase is typically homogenous over multi-Mb wide chromosomal regions called replication domains, and can change abruptly over tens of kb across the boundaries separating two domains 39. These replication domains typically encompass multiple contiguous TADs 12, and the early and late replicating domains, respectively, correspond to stretches of TADs belonging to the globally transcriptionally active genomic compartment (A-type 5) and the transcriptionally inert one (B-type) 40 – see Fig. 2E. The early or late nature of DNA replication is globally conserved during cell differentiation, but some regions of the genome can change their replication timing during cell differentiation 39, different regions changing timing in different cell types 41. Interestingly these shifts happen in a domain-wide fashion, at the Mb or sub-Mb scale. A recent report, focusing on two loci in the mouse, reported that some TADs can concomitantly shift in replication timing and in Hi-C genomic compartment, during cell differentiation 42. It is therefore tempting to speculate that such discrete changes in replication timing actually correspond to the replication timing shift of a single or a small group of contiguous TADs (Fig. 2E). It appears that chromatin folding into TADs could therefore also be the basis of the modularity in replication timing changes, and determine the extent of a region that undergoes a replication timing shift (Fig. 1E). This could now in principle be explored genome-wide and in multiple cell types. Importantly, this also suggests that chromosome segmentation into TADs may also play a role beyond the Mb, by acting as building blocks for higher-order chromosomal structures (see Box 1).

With all these considerations in mind, it seems that an intimate link exists between chromosome segmentation in TADs on the one hand, and various other domain-wide chromosomal features on the other, such as chromatin composition, nuclear positioning, and replication timing. Although these features can change with cell identity, segmentation into TADs is remarkably stable 12,13, raising the possibility that chromatin folding into TADs may actually provide, at least in some cases, a direct basis for domain-wide changes in various features of chromatin during development. Further investigation will clarify the mechanisms linking these aspects of chromosome organization.

Having described how TADs can be connected to various structural features of chromosomes, we will now discuss how they may also play a direct functional role in controlling the emergence and the activity of *cis*-regulatory landscapes.

## Transcriptional regulation

It is well established that the transcriptional activity of any given promoter is influenced by regulatory input coming from its chromosomal environment. In many instances remote control elements, such as distal enhancers, exert their regulatory effects across large genomic distances (up to hundreds of kb). This mechanism is thought to involve physical contact between these regulatory elements and their target promoters, which is permitted by the folding pattern of the chromatin fiber. Examples illustrating the diversity by which enhancers mechanistically stimulate promoter activity have been reviewed elsewhere 43, but many key aspects of this process remain enigmatic. How does an enhancer find its target amongst the many promoters that surround it? How can several distinct regulatory elements, sometimes scattered over hundreds of kb, all participate in the control of a given promoter? What is the extent of the chromosomal neighborhood involved in the control of a given promoter, and its possible dynamics during development? Below we discuss these issues in the context of the discovery of chromatin folding into TADs.

### TADs: A structural basis for regulatory landscapes?

TADs are, by definition, chromosomal regions within which DNA sequences most often establish long-range physical contact. This means that any given promoter will be contacted most frequently by sequences belonging to its TAD. It is therefore expected that enhancers, and other regulatory elements that act through physical contact with target promoters, should mainly impact the promoters of the same TAD. For these reasons it is tempting to speculate that the TAD of a given promoter embodies its regulatory domain, meaning the chromosomal region containing its *cis*-regulating elements. In support of this notion, most if not all of the previously reported cases of very long-range (>500 kb) functional enhancer-promoter communications are found to occur within TADs 44–45. These include paradigmatic loci such as *Sonic Hedgehog* and the *Lmbr1* intron (1 Mb away) 46.

### TADs as guides in the traffic of *cis*-regulatory information

Applications of high-throughput 3C, such as Hi-C and 5C, have revealed that promoters can be contacted by a large set of sequences within their TAD. What can seem surprising is that promoters are not specifically contacted by enhancers, in general – as defined genetically or according to their epigenomic signature 12–47 – no matter the activity state of these promoters or enhancers (active, poised, or inactive). Likewise, enhancers do not appear only to contact promoters. This means that a given promoter is typically not tethered to a given enhancer in a one-to-one relationship 47, but each of these elements is generally able to associate with (or at least transiently “visit”) large chromosomal domains 8–47. This is a very important observation that has far-reaching consequences for our understanding of enhancer-promoter communication.

**Figure 3:**
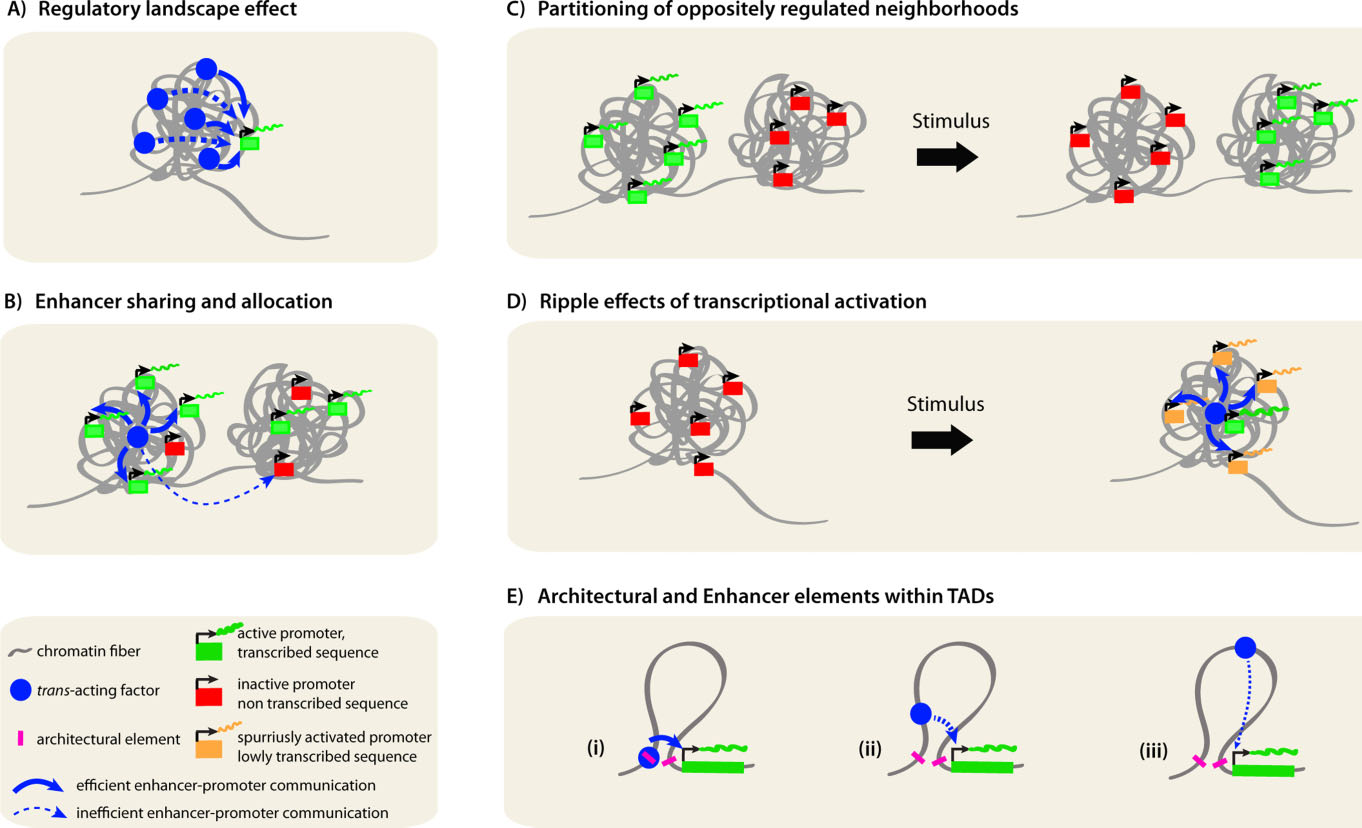
The link between TADs and domain-wide transcriptional regulation. Folding into TADs fosters long-range transcriptional regulation by allowing distal sequences to frequently contact each other. Folding into TADs allows A: multiple regulatory sequences to target the same promoter and conversely, B: multiple promoters to be targeted by a given regulatory element. C: Spatial partitioning segregates neighboring regulatory domains, allowing juxtaposed clusters to assume opposite transcriptional fates upon response to a stimulus. D: Activation of a regulatory element within a TAD can have minor yet measurable effects on secondary promoter targets, possibly explaining ripple effects of transcriptional activation. E: Elements controlling chromatin architecture or transcriptional activity can be distinct. When separate, architectural elements will control access of the transcription-controlling element to its target promoter, thereby playing an indirect but nonetheless integral role in the regulation of transcriptional activity.

Using state-of-the-art genetics and genomics approaches Duboule and colleagues 48 have recently revealed that a constellation of enhancers spread out over hundreds of kb are active in the same tissue and interact with the HoxD genes, leading the authors to propose the existence of a “regulatory archipelago” effect. This illustrates that a given promoter can be influenced by several distal regulatory elements within the same TAD (Fig. 3A). In another scenario these various enhancers can be active in distinct cell types. This is the case for the well-studied *Sonic Hedgehog* (*Shh*) locus, where various enhancers within the *Shh* TAD can be active in different tissues, depending on the *trans*-acting factors that bind and activate them 49.

The correlate of the regulatory archipelago effect is that a given enhancer will frequently visit several promoters within the same TAD (Fig. 3B). Sharing of regulatory elements within TADs could for example explain the broad yet sharply delimited radius of action of global control regions, such as the ones ensuring the co-expression of the various members of the two mouse *Iroquois* clusters or the proximal *HoxD/Evx2/Lnp* domain (for review see 50). We anticipate that the topological segmentation of chromosomes, by demarcating the chromosomal range that is accessible to an enhancer, both guides and constrains enhancer-promoter communication within TADs, thereby driving the preferential allocation of enhancers to the promoters that belong to the same TAD. Such an idea is supported by the situation at the mouse *HoxD* cluster, where proximal regulatory elements influence the proximal *HoxD* members. These lie together in the same TAD, but not with the distal *HoxD* members, which lie in a different TAD together with their own distal regulatory elements 12–48.

Enhancers are typically active in a tissue-specific fashion. How does this property relate to TAD dynamics during development and cell differentiation? Maybe surprisingly, TAD positioning along chromosomes remains largely invariant during cell differentiation 12–13. However, subtle but reproducible variations in the internal conformation of TADs occur, and these changes depend on the cell type. These key observations suggest that stable segmentation into TADs defines core landscapes, within which tissue-specific interactions can arise during the time-course of development, most likely depending on which *trans*-acting factors bind to the genomic elements involved in these connections. The mouse *HoxD* locus illustrates how various sets of enhancers, active in different tissues at different stages of development, all use the same core TAD architecture to convey their regulatory input in a cell-type specific fashion 48. In other cases, TAD folding may allow a common regulatory element to be used by several distinct promoters, depending on the developmental stage. The chromatin conformation inside the TAD or tissue-specific trans-acting factors expressed at a particular time, may ensure that only one promoter is engaged at a given time with the enhancers. Such developmental switching of promoter usage by a regulatory element is, for example, illustrated by the well-studied *β-globin* cluster, where the locus control region (LCR) and its targets lie in the same TAD (see 51 for review).

Based on this it appears that sharing of regulatory elements within TADs may in some cases lead to transcriptional coordination of genes located throughout Mb-wide chromosomal domains. TADs also facilitate the physical isolation of groups of genes and regulatory elements from their neighbors that assume different transcriptional dynamics upon response to a given stimulus (e.g. during cell differentiation, hormone response, etc. (Fig. 3C)). Such a phenomenon has been reported for the TADs that split the mouse *X-inactivation center* into several genomic domains which are oppositely regulated during early mouse ES cell differentiation 13.

Given the relative pervasiveness of chromosomal contacts within TADs, how can we explain that a given enhancer is able to find its specific promoter target? Actually, we would argue that there is little evidence showing that, in their native genomic context, distal enhancers are generally able to distinguish promoters in order to exert their control over a specific target. Rather, several lines of evidence suggest that within TADs, enhancers can exert broad regulatory effects that are often loosely targeted. A consequence of this is that rapid activation of control elements – for example during exposure to a given stimulus – can lead to extensive pervasive regulatory spills. This situation is exemplified by the ripple effects on transcriptional activation that are observed upon cell stimulation with growth-factors 52, where rapid transcriptional induction at one locus leads to activation of several neighboring promoters. We anticipate that promoters lying in the same TAD will tend to be affected by such ripple effects in situations when dynamic changes of transcriptional patterns occur, such as during stress or hormone response (Fig. 3D). This potential for co-regulation may be exploited in certain cases to ensure coordinated transcription patterns of clustered genes.

### Intra-TAD architecture and promoter-enhancer regulatory contacts

What is the structural basis for the communication between regulatory elements and promoters within TADs? A common textbook representation of enhancers is that they come into contact with promoters thanks to extensive looping out the intervening sequences. As recently discussed in depth by Fudenberg and Mirny 53, the detection of frequent contacts in a population of cells (i.e. peaks of 3C signal) actually does not necessarily imply the existence of such a looped-out configuration. This sort of signal can also be explained by the existence of a vast ensemble of chromatin configurations amongst the cell population, where the intervening DNA actually adopts compact but highly variable conformations without necessarily having to be extensively looped out 53. Such topological arrangements would actually agree better with FISH and polymer modeling data than looped-out configurations. In this situation intervening sequences are still excluded from the enhancer-promoter contact, but the overall conformation remains compact and, importantly, is very different from cell to cell. This has important consequences on how we envision transcriptional regulation. For example, it predicts extensive variability in local chromosome conformation, raising the question of how this relates to transcriptional variability and noise – possibly generating extensive “spatial effect variegation” (for review see 54) – either between cells or across time.

The apparent specificity that accompanies the preferential engagement of an enhancer with a given promoter may therefore rely on their inherent ability to maintain this contact, even temporarily, rather than to establish it. The sensitivity of 3C-based assays, which sample millions of cells, may reveal a tendency in the cell population but detection of an association does not necessarily reflect the existence of a stable chromosomal structure. This also implies that the structural mechanisms that control chromatin conformation, and the probability of enhancer-promoter encounters, act upstream of promoter-enhancer regulatory communication. It also means that genomic elements (and the factors bound to them) controlling chromatin architecture can be distinct from the factors involved in modulating the actual process of transcription (Fig. 3E).

Given these considerations, it might therefore be expected that disrupting folding in TAD, either by genomic alterations or changes in the epigenomic makeup, can lead to redirection of regulatory influences. Indeed, deleting a TAD boundary at the *Xist/Tsix* locus has been reported to impair spatial insulation, leading to regulatory bleed-through from one domain to the other and transcriptional mis-expression 13. Intriguingly, it has been reported that a small fraction of TAD boundaries are cell-type specific 12–14. We speculate that in these cases the gain or loss of TAD-boundary activity may be a strategy to expose promoters to new large regulatory landscapes during development, something that could be tested by deleting such facultative boundaries or replacing them by stable boundaries (see “Note added in proof”).

These considerations also predict that altering the internal organization of TADs that contain enhancers that do not have an intrinsic specificity for their promoter target should alter the orchestration of long-range regulation. A compelling example of this is that shuffling genomic organization at the *Fgf8* locus is sufficient to redirect underlying enhancers to different promoter targets 55. Another example is that tandem duplication within the TAD containing the proximal regulatory region of the mouse *HoxD* locus prevents distal regulatory elements from accessing and properly regulating the *HoxD* cluster, without creating a new TAD boundary 56.

Enhancers are not the only genomic elements that use chromatin architecture to convey regulatory information from afar. For example, some non-coding RNA loci also appear to rely on a similar process to modulate transcription. Human *HOTTIP* produces an RNA that partners with chromatin-modifying enzymes and regulates transcription of the portion of the *HOXA* locus that lies in the same TAD 57. Importantly, interfering with HOTTIP expression does not affect the topology of the locus, again suggesting that organization in TADs is controlled by mechanisms that appear to act upstream of transcription. One interesting case of a non-coding RNA concerns the Xist transcript, that can modify chromosome architecture by randomizing chromosomal contacts on the inactive X chromosome 13–58 – see Box 1.

Altogether these findings suggest that rather than being highly specific of a target promoter, enhancers should be considered as elements that can come into contact with a wide set of sequences, including but not limited to their target promoters, thanks to the underlying chromosomal architecture. Their radius of action would therefore be determined by the mechanisms that control three-dimensional chromatin organization. It is expected that long-range regulatory contacts within TADs will depend on the kinetics of chromatin diffusion. Determining the actual frequency and duration of these contacts (as discussed by 59), as well as the physical parameters that govern them 60, and how these integrate with the actual act of transcription and its enhancement, should shed important light onto the mechanisms of long-range transcriptional regulation.

Future work will be needed to elucidate the mechanistic rules that underlie how architectural elements control the probability of enhancer-promoter contacts (Fig. 3E). Understanding how architectural elements have participated in the evolution of phenotypic diversity by their broad impact on the flow of *cis*-regulatory information, and to what extent they are implicated in human disease are other topics of interest. This will be the subject of our last section.

### A link between chromosome folding and genome evolution

**Figure 4:**
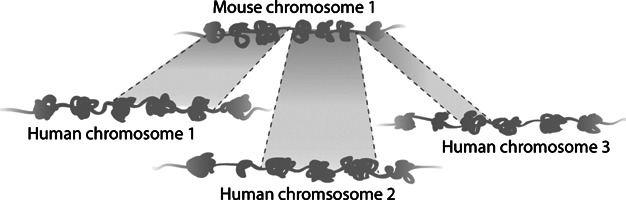
TAD-driven Mb-wide synteny. Synteny breaks within TADs would be expected to be counter-selected in general because they would disrupt underlying *cis*-regulatory connections. Such a phenomenon would lead to synteny of large chromosomal regions corresponding to groups of TADs, or even single TADs, with macrosynteny breaks occurring close to their boundaries. Expansion or retraction of these macrosyntenic regions can be observed, so that syntenic TADs or groups of TADs do not necessarily have the same genomic size in different species. The example shown here is illustrative.

It is well known that the presence of *cis*-regulatory elements lying a long way from their targets can lead to evolutionary constraints that would select against the breaking of their synteny 61. This results in maintenance of linkage for the whole regulatory block, including bystander loci that are not necessarily under the control of the underlying regulatory elements, simply because simple recombination cannot disentangle them from the rest of the domain 62. Given the observation that, at least in some cases, TADs provide a structural basis for such *cis*-regulatory landscapes 13, we anticipate that loci within the same TAD will have a tendency to be syntenic across species. Preliminary inspection of available data on conserved synteny blocks 63 supports this idea, and this could now be explored further quantitatively (Fig. 4). This phenomenon would also explain the overall conservation of the position of TAD boundaries between mouse and human 12. We hypothesize that such counter-selection of synteny breaks within TADs underlie a modular evolutionary pattern of the genome, where groups or TADs – or even single TADs – on a given chromosome in a given species would correspond to a similar arrangement on another chromosome in another species (Fig. 4). Cases of evident synteny loss within a TAD are also of interest as they may be linked to the appearance of species-specific expression patterns, whereby a single recombination event would have broken or reshuffled the underlying *cis*-regulatory landscape. Such a rearrangement would lead to dramatic and instantaneous rewiring of the *cis*-regulatory network. We therefore speculate that such “*cis*-ruption” 44 of TADs, when advantageous, may represent a mechanism of saltational phenotypic divergence (see 64–65 for insightful reviews on the theme of *cis*-regulatory evolution).

Such considerations also have implications for the genomic (or epigenomic) features that may define given sequences to act as TAD boundaries. For example, the observation that promoters of house-keeping genes are often found close to TAD boundaries has been proposed to reflect a possible mechanistic explanation for boundary formation and topological insulation. An alternative – and non-exclusive – hypothesis is that housekeeping genes would tend to become positioned close to TAD boundaries during evolution because their presence within TADs would interfere with their constitutive expression and might also perturb the proper functioning of long-range regulatory landscapes. The same reasoning holds for SINE elements or even CTCF binding sites, as well as for the reported tendency of P-element transgenes to insert close to TAD boundaries in *Drosophila* 14. One can envision several ways for testing these possibilities, such as removing a boundary-associated feature and measuring whether this disrupts topological insulation (as has been shown in one case at the *Xist/Tsix* locus 13); or else analyzing the effects of placing an ectopic feature (SINE, housekeeping gene, transgene, …) within a TAD on proper long-range regulation.

In the context of such considerations it seems highly likely that segmental folding of chromosomes has an impact on genome evolution, mainly because of the evolutionary pressure exerted by the *cis*-regulatory landscapes it helps to create. Mapping of TADs in diverse species will open the possibility to further explore this exciting avenue.

## Disrupting and exploiting TAD organization

### Experimentally challenging the hypothesis

How might one test whether chromosome segmentation in TADs actually underlies domain-wide chromatin states? What experiments could address the role served by such modularity in the definition of *cis*-regulatory domains? Approaches involving genome engineering to alter chromosome conformation would be one way of addressing such questions.

**Figure 5:**
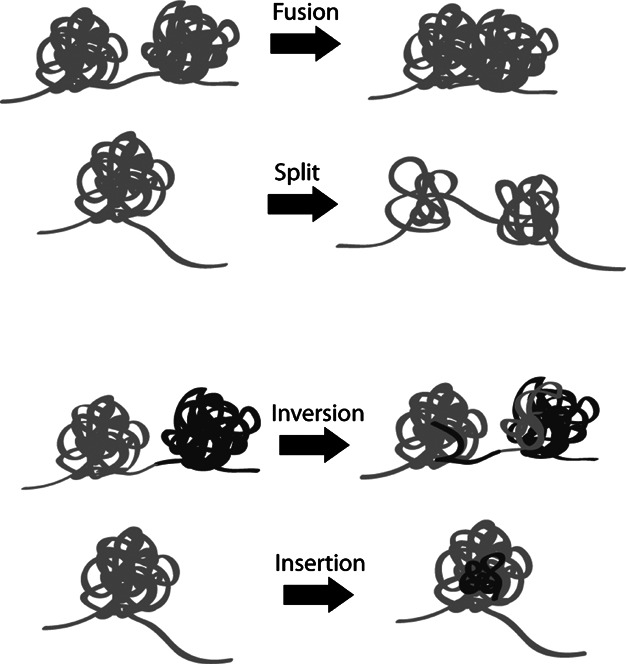
Experimental strategies to disrupt folding into TAD. Chromosome engineering could be used in various ways to alter TAD architecture and study the effects on domain-wide chromatin features and long-range regulation. For example, disrupting a TAD boundary separating two chromatin domains or inserting a boundary within a chromatin domain could be used to address the role of spatial organization in defining segmental chromatin blocks. Inversion around a TAD boundary or ectopic insertion within a TAD could be used to test to what extent DNA sequences are autonomous in setting up their chromatin state and to what extent they are influenced by the chromatin state of their TAD. The same experiments could be used to address the role that folding into TADs plays at the level of long-range transcriptional regulation.

First, disrupting folding in a TAD could provide an opportunity to address what happens to other domain-wide features of chromatin. For example fusing two neighboring TADs, which can be achieved by deleting the boundary between them (Fig. 5, 13), could be used to investigate the extent to which the structural partitioning of chromatin domains or “blocks” relies on their spatial segregation in distinct TADs. Conversely, splitting a TAD by targeted insertion of an ectopic boundary (Fig. 5) might be used to determine whether chromatin states at the different parts of a block rely on their physical clustering in a single TAD. If chromatin folding in TADs plays a role in the process, then chromatin states should be altered when TAD folding is disrupted. Such genomic rearrangements could also enable addressing the impact of TAD disruption on the long-range regulatory principles described in Fig. 2, for example by examining transcriptional activity at different stages of development or in response to different stimuli. We expect that disrupting enhancer-promoter contact by altering TAD organization should mimic their loss of function.

Another approach would be to study situations where genomic landscapes are reshuffled without affecting TAD organization. This would enable the testing of how autonomous a given genomic region is in setting up its chromatin make-up, as opposed to being influenced by its chromosomal neighborhood. Engineering large chromosomal inversions, for example using CRE/Lox-mediated recombination techniques 66,67, could be a way of symmetrically inverting large chromosomal neighborhoods around TAD boundaries. This would provide the opportunity to address how chromatin composition on each side of the inversion is affected (Fig. 5). Inserting large chromosomal segments within existing TADs, for example using bacterial artificial chromosome (BAC) transgenesis, could also be used to address how domain-wide features of chromatin are edified with respect to TADs. Such alterations of chromosome architecture could be specifically targeted around critical genic or regulatory sequences, to determine whether chromosomal architecture plays a direct role in modulating genic activity e.g. during development or stress response.

### Practical applications of TADs

Finally, we would like to consider the possible practical applications that chromatin folding into TADs can bring. Over the years genome-wide association studies (GWAS) have implicated many non-coding DNA sequence variants, such as single nucleotide polymorphisms or small copy number variants, as possible drivers of a wide range of traits, including a plethora of human diseases. Understanding how such variants lead to transcriptional mis-regulation and ultimately to a disease state is a great challenge. The first bottle-neck is to identify the gene target(s) that are transcriptionally mis-regulated when the driver sequence variants are present. If TADs can define *cis*-regulatory landscapes, then it is to be anticipated that the primary candidates for the relevant gene targets are those lying within the same TAD as the variant identified by GWAS. Given the stability of TAD positioning along chromosomes during development 12–13, it seems unnecessary to perform at first cell-type specific experiments. Such an analysis could in fact be guided by existing genome-wide data 12. We suggest that, in the same vein, the hunt for distal variants causing transcriptional mis-regulation of a disease-associated gene should first be performed in the TAD in which the gene in question lies, as this is the most likely genomic interval to harbor its long-range control elements.

Transgenesis approaches are frequently used to define gene function and expression patterns. This represents another practical application where knowledge of the segmental architecture of chromosome into TADs could be critical. In the context of our discussion of the implications of TADs for *cis*-regulatory landscapes, it would be expected that transgenes covering most of the TAD harboring a locus of interest would be the most likely to recapitulate appropriate gene expression. An illustration of this can be found with the *Xist/Tsix* locus, where single copy transgenes harboring only part of the *Xist* TAD or of the *Tsix* TAD are unable to recapitulate proper expression during development 13. The use of such information for predicting transgenes with accurate expression could be tested most easily in *Drosophila*, where TADs are of a smaller size, ∼100 kb on average, and for which libraries of TAD-sized transgenes are readily available.

Another important consideration is that flanking transgenes by TAD boundaries could likely insulate their expression from the influence of neighboring control elements and minimize position effects. Along the same lines, the choice of transgene insertion site is critical. Insertions of insulator-containing transgenes might disrupt TAD folding, as these sequences might act as ectopic boundaries, and disrupt the underlying regulatory landscape. Targeting transgene insertions close to endogenous TAD boundaries may therefore represent the safest strategy to house such transgenes.

## Conclusions and prospects

In this paper, we have discussed a series of hypotheses concerning the manner in which chromosome segmentation into TADs might relate to, and maybe even underlie, several aspects of chromatin architecture and metabolism, as well as long-range transcriptional regulation and genome evolution. This opens up several exciting avenues that could be explored in order to advance our understanding of chromosome structure and function. What are the molecular mechanisms that drive such folding patterns? What is the temporal fluctuation of this organization, in terms of chromatin motion, and how does this relate to the transcriptional dynamics of the underlying loci? What are the consequences of disrupting this organization, and do such alterations underlie diseases? Tackling these questions, using combinations of genomic engineering, genome-wide approaches as well as single-cell and live imaging approaches, with the ultimate aim of understanding the functional impact of chromatin architecture, represents an exciting challenge.

## Note added in proof, post peer review

A recent study at the *HoxD* locus has provided evidence that genes lying close to a TAD boundary can actually fold with either one of the two neighboring domains, in a tissue and developmental stage specific fashion. This folding shift was shown to provide a modular mechanism for HoxD exposure to two distinct regulatory landscapes during limb development 69.

## Abbreviations

BAC: bacterial artificial chromosome
3C: chromosome conformation capture
5C: chromosome conformation capture carbon copy
FISH: fluorescence in situ hybridization
GWAS: genome-wide association study
kb: kilobase
LAD: lamina-associated domain
LCR: locus control region
Mb: megabase
TAD: topologically associating domain
